# Clinical characteristics and outcome of fetuses with ventriculomegaly: a retrospective multicenter study

**DOI:** 10.1007/s00404-025-08279-x

**Published:** 2026-01-03

**Authors:** Ebru Alici Davutoglu, Bilge Çetinkaya Demir, Yasemin Doğan, Niyazi Cenk Sayın, Oya Demirci, Tuğba Saraç Sivrikoz, Ismail Yilmaz, Cihan İnan, Murad Gezer, Recep Has, Atıl Yuksel, Riza Madazli

**Affiliations:** 1 Department of Obstetrics and Gynecology, Division of Perinatology, Cerrahpasa Medical Faculty, Istanbul University-Cerrahpasa, Kocamustafapasa, St. No. 53, 34098 Istanbul, Turkey; 2 Department of Obstetrics and Gynecology, Division of Perinatology, Uludag Medical Faculty, Bursa Uludag University, Bursa, Turkey; 3 Department of Obstetrics and Gynecology, Division of Perinatology, Kocaeli Medical Faculty, Kocaeli University, Kocaeli, Turkey; 4 Department of Obstetrics and Gynecology, Division of Perinatology, Trakya Medical Faculty, Trakya University, Edirne, Turkey; 5 Perinatology Department, Zeynep Kamil Maternity and Child Hospital, Istanbul, Turkey; 6 Department of Obstetrics and Gynecology, Division of Perinatology, Istanbul Medical Faculty, Istanbul University, Istanbul, Turkey

**Keywords:** Fetal ventriculomegaly, Outcome, Genetic investigation, Counseling

## Abstract

**Purpose:**

To evaluate the incidence of associated structural anomalies, chromosomal/genetic abnormalities, infections and outcomes of fetuses with ventriculomegaly (VM).

**Methods:**

Retrospective multicenter cohort study of 627 pregnancies with fetal VM. VM was classified as mild, moderate, or severe and isolated or non‐isolated. Genetic, obstetric and outcome data were collected and compared according to VM categories.

**Results:**

The incidences of associated structural anomalies were 21.9%, 53.1% and 63.9% in mild, moderate and severe VM, respectively (*p* = 0.032 mild vs. moderate-severe). The incidences of genetic abnormality and fetal infection of the total VM group were 16.1% and 0.8%, respectively, with no significant differences between the VM categories (*p* > 0.05). The incidences of pathogenic genetic variant in the mild, moderate and severe VM were 13.5% (5/37), 16.7% (3/18) and 38.1% (8/21), respectively (*p* = 0.032 mild vs. severe). Fetal MRI identified additional CNS anomalies in 5.6% of cases. The incidences of surviving babies with neurological morbidities were significantly higher in fetuses with non-isolated VM groups than in isolated VM groups (*p* < 0.001).

**Conclusion:**

The prognosis of fetuses with VM mostly depends on the severity and the associated anomalies. In all types of fetal VMs additional genetic investigations are valuable.

**Supplementary Information:**

The online version contains supplementary material available at 10.1007/s00404-025-08279-x.

## Introduction

Fetal ventriculomegaly (VM) defined as an atrial diameter of the lateral ventricle of 10 mm or more on ultrasound, is the most frequently occurring central nervous system (CNS) abnormality in pregnancy with an estimated incidence of 0.3–2 per 1000 pregnancies [1]. Fetal VM can be subdivided into mild (10.0–11.9 mm), moderate (12.0–14.9 mm), and severe (≥ 15.0 mm) VM [2]. VM is easily recognized by ultrasound by measuring the atrial width, and measurement of the lateral cerebral ventricles is recommended as part of the routine anomaly scan [3]. Dilatation of the fetal cerebral ventricles is a generic sonographic sign that is common to several pathological entities carrying different prognoses. The prognosis depends on whether it is combined with structural abnormalities, chromosomal aberrations, congenital infections and the progression of the ventricular dilation [4–6].

Following confirmation of the diagnosis of fetal VM, a thorough evaluation, including complete examination of the fetal anatomy, detailed neurosonographic assessment, consideration of fetal magnetic resonance imaging (MRI), amniocentesis for karyotype and chromosomal microarray analysis (CMA), and investigations for fetal infections should be performed [6, 7]. Recently, single-nucleotide variant (SNV) analysis via exome sequencing (ES) has been introduced and found to be valuable in the assessment of fetuses with fetal VM [5, 8].

Due to the numerous contributing factors, prenatal counseling of parents with a fetus diagnosed with VM is very challenging because of the uncertainty of neurodevelopmental outcome and the decision for termination of pregnancy (TOP). In this retrospective multicenter study, we assessed the incidence of associated CNS and non-CNS anomalies, chromosomal/genetic abnormalities, infectious causes, and outcomes of fetuses with prenatally diagnosed VM, and compared the results according to the subgroups of VM. And also, aimed to identify the risk factors for abnormal outcome in order to improve the quality of prenatal counseling.

## Methods

This is a retrospective multicenter cohort study involving six tertiary Perinatology Departments (Cerrahpasa, Istanbul, Kocaeli, Trakya and Uludag Medical faculties and Zeynep Kamil Maternity hospital) in the Marmara region of Turkiye. The study population included 627 pregnancies with prenatally diagnosed fetal VM from January 01, 2014 to September 01, 2024. Neural tube defects, twin pregnancies and cases with incomplete follow-up were excluded. All ultrasonographic examinations were done by perinatologists, including multiplanar neurosonography using transabdominal and/or, when feasible, transvaginal approach, as suggested by the ISUOG guidelines [3]. Multidisciplinary evaluation of the cases by perinatologist, geneticists and pediatric neurologists were applied when necessary. The study was approved by the local Ethical Committee of Cerrahpasa Medical Faculty (E-74555795-050.04-1249111). VM was categorized into mild, moderate, or severe based on the measurements on the first prenatal ultrasound performed as follows mild VM (10.0–11.9 mm), moderate VM (12.0–14.9 mm) and severe VM (≥ 15.0 mm). Unilateral VM was defined as one lateral ventricle with an atrial measurement of 10.0 mm or more, while bilateral VM was defined as both lateral ventricle atrial measurements ≥ 10.0 mm. The fetuses were also classified as “isolated ventriculomegaly” if no associated structural anomaly was detected, with negative serology and genetic prenatal diagnosis. Fetal MRI was performed to 247 pregnancies to confirm the diagnosis and to evaluate the fetal brain for additional anomalies. Indication for fetal MRI was given by the individual physicians without a specific standard or criterion for its use. These women did not have any known or suspected contraindications to MRI and agreed to provide written consent after full explanation. Data of these MRI findings were collected and additional radiological abnormalities were recorded.

Invasive prenatal diagnostic tests for genetic abnormalities were performed to 322 pregnancies via amniotic fluid sampling, cord blood sampling and chorionic villus sampling. Conventional karyotype analysis, CMA and whole exome sequencing (WES) was performed in 322, 173 and 76 cases, respectively, and data from these genetic tests were collected. Maternal infectious serology (such as Toxoplasmosis, Cytomegalovirus, Rubella virus, Treponema pallidum) and, in some cases, amniotic fluid assessment by PCR for perinatal infections were also collected.

Maternal age, gestational age at the time of diagnosis, CNS and non-CNS anomalies, gestational week at delivery, birth weight and postnatal definitive diagnosis were analyzed. Outcome parameters were live births, TOP, intra‐uterine fetal demise, neonatal and infant death, alive at > 6 months with and without morbidity. The decision of TOP was made by the official ‘Termination of Pregnancy Council’ of the involved centers according to national laws. Neonatal death was defined as death within the first 28 days of life, and infant death was defined as death within the first year. The mean age of the surviving neonates was 4.1 ± 3.2 years (range, 6 months–10 years) at the time of the study. Data on surviving infants were obtained by telephone interviews with the parents enquiring about major neurologic abnormalities, shunting operations and epilepsy medications. Major neuromotor abnormalities (cerebral palsy, spastic hemi or quadriplegia, major intellectual disability) epilepsy and ventriculoperitoneal shunt operations due to VM was defined as morbidity. Outcomes of the mild, moderate and severe VM groups were analyzed and compared.

The data were analyzed using Statistical Package for Social Sciences software version 20.0 for Windows (SPSS Inc., Chicago, IL). Descriptive statistics were used to analyze demographic data and clinical characteristics in the three defined VM subgroups. Categorical variables were reported as frequencies and percentages, with mean and standard deviation reported for continuous variables. The distribution of numerical variables was analyzed using the Shapiro–Wilk test. Continuous variables with normal distribution were analyzed with an independent two-sample t-test and one-way ANOVA, while the Kruskal–Wallis and Mann–Whitney U-test tests were applied for variables that did not follow a normal distribution. Categorical variables were compared through Fisher’s exact/ Chi-square tests.

## Results

Of the 627 VM cases, 306 (48.8%) were classified as mild, 113 (18%) as moderate, and 208 (33.2%) as severe VM. The clinical characteristics and the outcomes of the study groups are shown in Table 1. The mean maternal age, mean gestational age at the first examination, mean gestational age at delivery and mean birthweight of the total group was 29.4 ± 5.5 years, 24.9 ± 5.4 weeks, 37.3 ± 2.7 weeks and 3011 ± 703 g, respectively. No significant differences in mean maternal age, gestational age at first examination, gestational age at delivery and birthweight were identified between the VM categories (*p* > 0.05). Regarding fetal characteristics, isolated and unilateral VM were more prevalent in mild than moderate—severe VM (*p* < 0.001). The incidences of an associated structural anomalies were 21.9%, 53.1% and 63.9% in mild, moderate and severe VM, respectively (*p* = 0.032, mild vs. moderate-severe). The incidences of genetic abnormality and fetal infection of the total VM group were 16.1% and 0.8% respectively, with no significant differences between the VM categories (*p* > 0.05) (Table 1).

**Table 1:** The clinical characteristics and the outcomes of the ventriculomegaly subgroups

	Mild	Moderate	Severe	Total
	VM	VM	VM	
*N*	306 (48.8)	113 (18)	208 (33.2)	627 (100)
Maternal age (years)	29.9 ± 5.6	29.1 ± 5.3	28.8 ± 5.5	29.4 ± 5.5
Gestation age at examination (weeks)	24.7 ± 5.0	24.5 ± 5.7	25.6 ± 5.7	24.9 ± 5.4
< 20 weeks	47 (15.4)	23 (20.4)	28 (13.5)	98 (15.6)
20–24 weeks	109 (35.6)	37 (32.7)	74 (35.6)	220 (35.1)
25–32 weeks	130 (42.5)	41 (36.3)	78 (37.4)	249 (39.7)
> 32 weeks	20 (6.5)	12 (10.6)	28 (13.5)	60 ( 9.6)
Laterality				
Bilateral VM	147 (48)	93 (82.3)	197 (94.7)	437 (69.7)
Unilateral VM	159 (52)	20 (17.7)	11 (5.3)	190 (30.3)
Isolated	223 (72.9)	49 (43.4)	65 (31.3)	337 (53.7)
Associated structural anomalies	68 (22.2)	60 (53.1)	133 (63.9)	261 (41.6)
Central nervous system	50 (16.3)	47 (41.6)	120 (57.7)	216 (34.4)
Non-central nervous system	18 (5.9)	13 (11.5)	13 (6.3)	44 (7.1)
Genetic abnormality (*N* = 322)	18/149 (12.1)	13/61 (21.3)	21/112 (18.7)	52/322 (16.1)
Fetal infection	2 (0.6)	1 (0.9)	2 (0.9)	5 (0.8)
Gestational age at birth (weeks) (*N* = 455)	37.6 ± 2.5	37.4 ± 2.6	36.6 ± 3.1	37.3 ± 2.7
Birthweight (grams) (*N* = 455)	3038 ± 645	3045 ± 732	2934 ± 797	3011 ± 703
Termination of pregnancy	40 (13.1)	35 (30.9)	85 (40.9)	160 (25.5)
Intrauterine death	2 (0.7)	4 (3.5)	6 (2.9)	12 (1.9)
Neonatal and infant death	16 (5.2)	9 (7.9)	25 (12)	50 (7.9)
Alive at > 6 months of age	248 (81.1)	65 (57.5)	92 (45.7)	405 (64.6)
Without morbidity	233/248 (93.9)	53/65 (81.5)	38/92.4 (1.3)	324/405 (80)
With morbidity	15/248 (6.1)	12/65 (18.5)	54/92 (58.7)	81/405 (20)

Data are expressed as mean ± sd, *n*, (%) and *n*/*N*, (%)

*VM* ventriculomegaly

Concerning the outcomes of the study populations, the incidences of TOP in mild, moderate and severe VM were 13.1%, 30.9%, and 40.9% respectively, with a significantly higher incidence of termination in severe VM (*p* < 0.001). Incidences of intrauterine and neonatal and infant death of pregnancies with mild, moderate and severe VM were 0.7% and 5.2%, 3.5% and 7.9% and 2.9 and 12%, respectively. Incidences of intrauterine death were significantly lower in mild VM and neonatal and infant death were significantly higher in severe VM than in the other VM categories (*p* < 0.05). Of the 248 babies with mild, 65 babies with moderate and 92 babies with severe VM alive at > 6 months of age, 15 (6.1%), 12 (18.5%) and 54 (58.7%) were with neurological morbidity, respectively (Table 1).

Distribution of associated CNS anomalies according to the type of VM is illustrated in Table 2. The incidences of CNS anomalies were 16%, 41.6% and 57.7% in mild, moderate and severe VM, respectively (*p* < 0.001, mild vs. moderate-severe). Corpus callosum agenesis was the most frequent CNS anomaly detected in all of the VM categories (Table 2).

**Table 2:** Distribution of associated central nervous system anomalies according to the type of ventriculomegaly

	Mild VM	Moderate VM	Severe VM
*N*	50/306 (16.3)	47/113 (41.6)	120/208 (57)
Corpus callosum agenesis	33	25	55
Dandy Walker	7	6	11
Intracranial hemorrhage	2	4	13
Intracranial cyst	3	3	6
Lissencephaly	3	1	4
Porencephaly	1	–	1
Encephalocele	1	2	2
Cerebellum hypoplasia	–	1	12
Semilober/Lober holoprosencephaly	–	3	2
Blake pouch cyst	–	1	1
Intracranial tumor	–	–	4
Hemimegalencephaly	–	–	1
Schizencephaly	–	–	2
Hydranencephaly	–	–	2
CSP agenesis	–	1	2
Walker Warburg syndrome	–	–	1
Galen vein aneurysm	–	–	1

Data are expressed as *n*, (%) and *n*/*N*, (%)

*VM* ventriculomegaly

Fetal MRI was performed in 247 (39.4%) of the fetuses and the mean gestational age at fetal MRI examination was 27.9 ± 4.3 weeks (range, 20–37 weeks). Rates of additional anomalies detected on prenatal MRI are shown in Table 3. There were 14 (14/247, 5.6%) additional CNS anomalies diagnosed by fetal MRI in our series. Incidences of additional anomalies detected on prenatal MRI were 4.5%, 6.7% and 6.4% for mild, moderate and severe VM, respectively, without any significant difference between the VM categories (*p* > 0.05) (Table 3).

**Table 3:** Additional anomalies diagnosed on prenatal magnetic resonance imaging

	Mild VM	Moderete VM	Severe VM
Fetal MRI	110/306 (35.9)	60/113 (53.5)	77/208 (37.1)
Gestational age at examination (weeks)	27.8 ± 3.9	26.8 ± 4.6	28.6 ± 4.4
Additional anomaly	5/110 (4.5)	4/60 (6.7)	5/77 (6.4)
Complete corpus callosum agenesis	1	1	1
Partial corpus callosum agenesis	1	1	–
Hemorrhage	1	1	1
Lisencephaly	2	–	–
Walker Warburg syndrome	–	–	1
Cerebellum hypoplasia	–	–	1
Intracranial tumor	–	–	1
Lober holoprocencephaly	–	1	–

Data are expressed as *n*/*N*, (%) and *n*, (%)

*VM* ventriculomegaly

Genetic analysis was performed in 322 (51.4%) of the cases; in all cases karyotype analysis was performed, in 173/322 (53.7%) cases CMA, and in 76/322 (23.6%) cases additional WES. Invasive prenatal diagnostic tests for genetic abnormalities were performed via amniotic fluid sampling, cord blood sampling and chorionic villus sampling in 268 (83.3%), 38 (11.8%) and 16 (4.9%) fetuses, respectively. Distribution of genetic abnormalities according to the type of VM is illustrated in Table 4. The incidences of karyotype anomalies were 6.1% (9/149), 14.8% (9/61) and 6.3% (7/112) in mild, moderate and severe VM, respectively, which was not significantly different between the subgroups (*p* > 0.05). Trisomy 21 was the most frequent karyotype anomaly detected in all VM categories. The incidences of copy number variants (CNVs) detected by CMA in the mild, moderate and severe VM were 5.3% (4/75), 3.2% (1/31) and 8.9% (6/67), respectively, without any significant difference between the subgroups (*p* > 0.05). The incidences of pathogenic or likely pathogenic genetic variant detected by WES in the mild, moderate and severe VM were 13.5% (5/37), 16.7% (3/18) and 38.1% (8/21), respectively (*p* = 0.032 mild vs. severe). Of the 52 fetuses with a genetic abnormality, 22 (42.3%) had no additional structural anomaly. The incidences of genetic abnormalities in fetuses with and without additional structural anomalies were 18.1% (29/161) and 14.2% (23/161), respectively. The incidences of karyotype anomalies, CNVs and pathogenic genetic variant in fetuses without structural anomalies were 5.1% (5/98), 10.7% (3/28) and 10.3% (3/29) for mild, 8.3% (2/24), 11.1% (1/9) and 0% (–/10) for moderate and 5.1% (2/39), 27.3% (3/11) and 50% (4/8) for severe VM, respectively (Table 4).

**Table 4:** Distribution of genetic abnormalities according to the type of ventriculomegaly

	Mild VM	Moderete VM	Severe VM
Genetic analysis	149/306 (48.7)	61/113 (53.9)	112/208 (53.8)
Karyotype anomalies (*N* = 322)	9/149 (6.1)	9/61 (14.8)	7/112 (6.3)
Trisomy 21	6	5	4
Trisomy 18	1	–	2
Trisomy 13	1	1	1
Trisomy 22	–	1	–
Triploidy	1	2	–
Copy number variants (*N* = 173)	4/75 (5.3)	1/31 (3.2)	6/67 (8.9)
Pathogenic genetic variant (*N* = 76)	5/37 (13.5)	3/18 (16.7)	8/21 (38.1)
Without structural anomaly			
Karyotype anomalies (*N* = 161)	5/98 (5.1)	2/24 (8.3)	2/39 (5.1)
Copy number variants (*N* = 48)	3/28 (10.7)	1/9 (11.1)	3/11 (27.3)
Pathogenic genetic variant (*N* = 47)	3/29 (10.3)	–/10 (–)	4/8 (50)

Data are expressed as *n*/*N*, (%) and *n*, (%)

*VM* ventriculomegaly

There were five fetal infections confirmed by amniotic fluid PCR analysis: three cases with CMV and two cases with toxoplasma infections. There were two TOP due to fetal infections; one fetus with toxoplasma had severe VM, periventricular nodule and hyperechogenic bowel and the other fetus with CMV had moderate VM, periventricular calcification and microcephaly. One of the two fetuses with CMV infections that were born alive had neonatal death and the other one is alive with morbidity. One case of congenital toxoplasma infection is alive without morbidity.

The outcomes of isolated and non-isolated mild, moderate and severe VM groups are shown in Table 5. The incidence of isolated VM in our study group was 53.7% (337/627). Of the 337 fetuses with isolated VM, 27 with severe VM opted for TOP. The incidences of fetuses alive at > 6 months of age were 96.8%, 95.9% and 49.2% for mild, moderate and severe isolated VM, respectively. The rates of fetuses alive at > 6 months of age with neurological morbidity were 1.9%, 8.5% and 43.7% for mild, moderate and severe isolated VM, respectively. The incidence of surviving babies with neurological morbidity was significantly higher in fetuses with severe isolated VM than mild and moderate VM (*p* < 0.001). The rates of fetuses alive at > 6 months of age with neurological morbidity were 34.4%, 44.4% and 66.7% for mild, moderate and severe non-isolated VM, respectively. The incidences of surviving babies with neurological morbidities were significantly higher in fetuses with non-isolated than isolated VM groups (*p* < 0.001) (Table 5).

**Table 5:** The outcomes of isolated and non-isolated mild, moderate and severe ventriculomegaly groups

	Mild VM		Moderate VM		Severe VM	
	İsolated	Non-isolated	Isolated	Non-isolated	Isolated	Non-isolated
*N*	223	83	49	64	65	143
TOP	–	40 (48)	–	35 (54.7)	27 (41.5)	58 (40.6)
Intrauterine death	1 (0.4)	1 (1.2)	1 (2)	3 (4.7)	–	6 (4.2)
Neonatal and infant death	6 (2.7)	10 (12.2)	1 (2)	8 (12.5)	6 (9.2)	19 (13.3)
Alive at > 6 months of age	216 (96.8)	32 (38.5)	47 (95.9)	18 (28.1)	32 (49.2)	60 (41.9)
Without morbidity	212 (98.1)	21 (65.6)	43 (91.5)	10 (55.6)	18 (56.3)	20 (33.3)
With morbidity	4 (1.9)	11 (34.4)	4 (8.5)	8 (44.4)	14 (43.7)	40 (66.7)

Data are expressed as *n*, (%)

*VM* ventriculomegaly, *TOP* termination of pregnancy

## Discussion

Incidences of mild, moderate and severe fetal VM in our study population are similar to those reported in the literature [1, 4, 5, 9]. Nearly half of the cases (48.8%) were mild VM in our study. The incidences of mild VM were 50.4% and 47.5% in the Chang et al. [1] and Moens et al. [5] series, respectively. In our study population, 50.7% of the fetal VM cases were diagnosed before 24 weeks’ gestation. In two other studies, the reported incidences of diagnosis before 24th gestational weeks’ were 60% [10] and 69% [5]. There were no significant differences in mean maternal age, gestational age at first examination, gestational age at delivery and birthweight between the VM categories in our study, in accordance with previous studies [5, 9]. Regarding fetal characteristics, isolated and unilateral VM were more prevalent in mild than moderate-severe VM, which is similar with other studies [1, 5, 9].

The overall rate of associated structural abnormalities was 41.6% in our study. It is well established that VM is associated with additional structural abnormalities, with a reported incidence ranging between 10 and 76% [1, 5, 9, 11]. Reported incidences of CNS and non-CNS malformations were 45% and 37.1% in Moens et al. [5] and 31.9% and 15.5% in Ryan et al. studies, respectively [9]. CNS malformations are more frequently reported in cases of moderate-severe VM ranging from 39 to 65%, than mild VM (10–76% with an average value of 41%) in the literature [1, 5, 9, 11]. In accordance with the literature, the incidence of CNS anomalies was significantly lower in mild than moderate-severe VM in our population. Corpus callosum agenesis was the most frequent CNS anomaly detected in all of the VM categories, consistent with previous reports [4, 5, 12]. It is highly recommended that a detailed anatomical and neurosonographic examination of the fetus should be performed by a perinatologist after the diagnosis of VM due to the high incidence of associated structural anomalies [13].

Fetal brain MRI has been accepted as a complementary method to improve diagnostic accuracy for brain abnormalities in fetal VM [13, 14]. The largest prospective MERIDIAN study reported that fetal MRI could detect additional CNS anomalies in about 23% of fetal VM cases [15]. In a recent systematic review, Di Mascio et al. found that, in fetuses with isolated mild and moderate VM, the incidence of CNS anomalies detected exclusively on MRI was 5% when dedicated neurosonography was performed and 16.8% with standard assessment of the fetal brain [16]. In a multicenter, retrospective, cohort study designed by the ENSO Working group demonstrated that in fetuses with isolated mild or moderate VM, the incidence of an associated fetal anomaly detected by fetal MRI was 5.4% [17]. The most frequent CNS anomalies detected by fetal MRI were cortical developmental malformations, intracranial hemorrhage and midline anomalies in reported series [16, 17]. In our study, MRI provided additional information in 4.5%, 6.7% and 6.4% for mild, moderate and severe VM, respectively, and 5.6% overall. Fetal MRI may be considered after detailed neurosonography and the integration of the different imaging techniques may improve the diagnosis and counseling in selected cases.

In utero infections, especially toxoplasmosis and cytomegalovirus, could be a cause of VM with a reported incidence of less than 5% [13]. There were five fetal infections (three CMV and two toxoplasma) confirmed by amniotic fluid PCR analysis in our study population, with an incidence of 0.8%. The incidences of fetal infections were not significantly different between the VM subgroups in our series. Associated ultrasonographic signs in the case of VM due to infection include the presence of microcephaly, irregular and hyperechogenic ventricular walls, hyperechogenic periventricular conglomerates, intraventricular synechiae and periventricular cysts [18]. Of the five fetuses with fetal infections three (two CMV and one toxoplasma) fetuses had ultrasonographic signs of fetal infection in our population.

Fetal VM is frequently associated with underlying genetic disorders [19]. In general, the incidence of genetic abnormality varies depending on the study population, atrial width, and association with other structural malformations [4, 13]. The genetic abnormality rate in our population was 16.1%, in which the rates were 14.2% and 18.1% for fetuses without and with associated structural anomalies, respectively. The reported incidences of chromosomal/genetic abnormalities in fetal VM without and with structural anomalies vary between 2.5 and 10% and 10–36%, respectively, in the literature [4, 5, 9, 20, 21]. The presence of associated abnormalities is an important factor that influences the rate of genetic abnormalities, thus potentially impacting the prognosis. Trisomy 21 was the most frequent karyotype anomaly detected in our cohort in accordance with the literature [19, 22]. The incidences of karyotype anomalies in fetuses without associated structural anomalies were 5.1%, 8.3%, and 5.1%, for mild, moderate and severe VM, respectively, in our study, which was not significantly different between the VM subgroups. The reported rate of karyotype anomalies in the case of fetal VM without structural anomalies ranges from 3 to 5% similar to our findings [13, 19]. The relationship between the degree of VM and the incidence of chromosomal abnormalities is conflicting in the literature and large-scale studies are still needed to determine the exact relationship [13].

Clinically significant CNVs are submicroscopic deletions and duplications, generally sized 1 kb (kilobases) or greater, that cannot be diagnosed using standard karyotyping and microarray analysis is needed for diagnosis [19]. There are more than 200 well-described microdeletion-duplication syndromes and CNVs are known causes of a broad spectrum of neurodevelopmental disorders and congenital malformations [19, 23]. In fetuses with VM, overall detection rates of CNVs vary between 5 and 10% of cases with a normal karyotype [19]. The incidence of CNVs in our cohort was 6.4% (11/173), in line with the literature. The rates of microdeletions were not significantly different between the VM subgroups in our cohort. Recently, diagnostic testing with CMA is mostly advised and became a common practice, when VM is detected [19, 24].

VM has also been described to be associated with single-gene disorders and more than 100 genes have been identified [19]. The rate of pathogenic findings on WES in fetuses with CNS anomalies are reported within a wide range from 3 to 55% in the literature [25]. A systematic review and meta-analysis by Mustafa et al., found that the rate of pathogenic or likely pathogenic ES was 45% in 117 cases of bilateral severe VM [26]. This rate was 35% in isolated cases, 38% in those with additional intracranial anomalies, and 54% in those with additional extracranial anomalies with bilateral severe VM [26]. In another meta-analysis involving 538 cases of postnatal congenital hydrocephalus, the incidence of pathogenic or likely pathogenic ES was 37.9% in all cases, 76.3% in consanguineous marriages and 21.3% in isolated and nonsyndromic cases [27]. The rate of pathogenic genetic variants in our series was 21.1% (16/76) overall. The incidences of pathogenic genetic variant for mild and severe VM without associated structural anomalies were 10.3% and 50%, respectively, where WES was performed in our cohort. Single-gene disorders were the most frequent genetic abnormality in severe VM in our study. Despite becoming more accessible, WES still remains fairly cost-intensive and time-intensive. However, WES analyses could be considered especially in severe VM, fetuses with associated structural anomalies and cases with other factors associated with mendelian inheritance, such as consanguinity and positive family history.

The overall outcome of fetal VM is dependent on the presence of associated abnormalities and the severity of VM [7]. Termination of pregnancy is reported to be frequently chosen by patients with non-isolated VM [4, 10, 28]. The incidences of TOP in non-isolated mild (48.8%), moderate (54.7%) and severe (40.6%) VM in our series are in line with previous studies [4, 28]. Early diagnosis of associated malformations, the width of the ventricular dilatation and the religious beliefs of the parents affect the decision of TOP. Incidences of surviving babies with neurological morbidities were significantly higher in fetuses with non-isolated VM than isolated VM as expected and similar to previous studies [4, 5, 10].

The survival rates of isolated mild and moderate VM were 97% and 96%, respectively, in our study, in accordance with previous reports [1, 4, 9]. Incidences of neurological morbidity of surviving fetuses with mild and moderate isolated VM were 1.9% and 8.5%, respectively, is also in line with other studies [20, 29, 30]. Studies evaluating neurological outcome of isolated mild-moderate VM have been limited by inconsistent definitions of neurodevelopmental delay, use of different scales for neurodevelopmental assessment, and inclusion of children at different ages, thus leading to wide variation in the reported incidence of neurodevelopmental delay [13]. In a recent meta-analysis of unilateral mild and moderate VM, the overall prevalence of abnormal neurodevelopment was reported to be 5.9% [29]. The nationwide registry-based study in Denmark, demonstrated a 5.6% neurodevelopmental delay in fetuses with parentally diagnosed isolated mild VM [30]. Although the neurodevelopmental outcome of children with postnatally confirmed isolated mild or moderate VM is generally favorable, neurodevelopmental disorders are slightly higher than in the general population [13].

Isolated severe VM is associated with poor neurodevelopmental outcome and high incidences of TOP [31, 32]. Of the severe isolated VM fetuses in our study population, 49.2% were alive at > 6 months of age and 44% of them had neurological morbidity. In a meta-analysis by Carta et al. demonstrated that the survival rate was 88% in isolated severe VM after excluding the TOP cases and of the surviving babies 18.6% had mild-moderate and 39.6% had severe neurologic, motor, and cognitive impairment [30]. In another recent meta-analysis by Ali et al., incidences of intrauterine fetal demise and infant mortality were 24.3% and 17.1%, respectively, in isolated severe VM and the incidence of adverse neurodevelopmental outcome in surviving fetuses was 58.4% [32].

Although the sample size of this study is quite big, there are some limitations that should be acknowledged. İt is a retrospective multicenter study, that possesses difficulties in data collection. The rate of genetic analysis for fetuses with and without additional structural anomalies were 61.7% (161/261) and 43.9 (161/366), respectively, which is low and may have bias in itself. Furthermore, WES was performed only 12.1% (76/627) of the cases, which is very low and may not reflect the real state. A notable proportion of pregnancies in the severe isolated VM group was terminated, thus not enabling the natural progression of these pregnancies. Additionally, the information collected about postnatal follow-up through phone interviews with parents, particularly regarding the lack of information from pediatric neurology follow-up, may diminish the reliability of our data.

## Supplementary Information

Below is the link to the electronic supplementary material.

Supplementary file1 (DOCX 14 KB)

## Data Availability

The data used to support the findings of this study are available from the corresponding author upon reasonable request.
