# Supplementary material for: Clinical characteristics and outcome of fetuses with ventriculomegaly: a retrospective multicenter study
Source: Arch Gynecol Obstet. 2026 Jan 3;313(1):7. doi: 10.1007/s00404-025-08279-x (PMC12764503; doi:10.1007/s00404-025-08279-x)
Supplement: Supplementary file 1 — Supplementary file1 (DOCX 14 KB) [file 404_2025_8279_MOESM1_ESM.docx]

Table : The list of the details of the genetic abnormalities in the study

Mild VM Moderete VM Severe VM

**Aneuploidies (N=25)** N=9 N=9 N=7

Trisomy 21 6 5 4

Trisomy 18 1 - 2

Trisomy 13 1 1 1

Trisomy22 - 1 -

Triploidy 1 2 -

**Copy number variants (N=11)** N=4 N=1 N=6

2q37.3 del, 7q35 del, 14q13.2 dup 1

9p24.3p22.3 and 3q26.1q29 dup 1

6q26q27 del, 1p34.1 dup 1

16q24.3 del 1

1q21.2 dup 1

18p11.31p11.23 dup 1

arr(GRCh37) 18q11.2q12.1 1

arr(GRCh37) 8p23.q24.3 1

arr(GRCh37) 17q25.3 1

4 p del 1

4q22.2 del, 5p13.2 dup 1

**Pathogenic genetic variant (N=16)** N=5 N=3 N=8

FGD1 homozygous 1

MAP2K1 heterozygous 1

PACS1 homozygous 1

BBS12 heterozygous 1

SOX2 heterozygous 1

WDR81 homozygous, PIK3R2 heterozygous 1

TCF4 and FUZ heterozygous 1

PUF60 heterozygous, WASHC5 heterozygous 1

POMT1 homozygous 1

FHL1 homozygous 1

LZTR1 heterozygous 1

KIF1A heterozygous 1

EP300 heterozygous, SON heterozygous, IL2RG homozygous 1

CHRNE homozygous, TSC2 heterozygous, DLL1 heterozygous 1

PTPN11 heterozygous 1

CHD7 heterozygous 1
